# Acacetin-loaded microemulsion for transdermal delivery: preparation, optimization and evaluation

**DOI:** 10.1080/13880209.2023.2207597

**Published:** 2023-05-10

**Authors:** Yajing Wang, Qian Chen, Xianfeng Huang, Xiaojing Yan

**Affiliations:** a Department of Pharmacy, Changzhou University, Changzhou, PR China; b Changzhou Key Laboratory of Human Use Experience Research & Transformation of Menghe Medical School, Changzhou Hospital affiliated to Nanjing University of Chinese Medicine, Changzhou, PR China

**Keywords:** Emulsions, solubility, simplex lattice experiment design, permeation enhancers, pharmacokinetics

## Abstract

**Context:**

Acacetin is reported as a potential drug candidate for the treatment of atrial fibrillation. However, clinical applications are limited by poor water solubility, limited ethanol solubility, and extremely low oral bioavailability.

**Objective:**

The present study prepared and evaluated acacetin-loaded microemulsion (ME) to achieve efficient pharmacokinetics together with no or minimal invasiveness for transdermal delivery.

**Materials and methods:**

The formulation of ME was determined by the water titration method based on solubility results. The optimized formulation was achieved by the simplex lattice experiment design. The optimized ME formulations FA, FB and FC (FA with 10% and 50% DMSO as enhancers, respectively) were evaluated by *ex vivo* permeation with Franz diffusion cell and excised mice skin. *In vivo* pharmacokinetic studies were also performed at 8 mg/kg in rats within 6 h by transdermal administration.

**Results:**

The optimal ME (FA) was comprised of 12.2% caprylic acid decanoate monoditriglyceride (MCF-NF), 39.8% Smix (RH40: Trans = 2:1 w/w) and 48% water, respectively. Acacetin-loaded FA with particle size 36.0 ± 3.6 nm and drug solubility 803.7 ± 32.1 mg/g was prepared. FB had significantly higher cumulative amounts and higher AUC_0-∞_ (196.6 ± 11.0 min × μg/mL, *p* < 0.05) than that FA alone (121.4 ± 33.1 min × μg/mL).

**Discussion and conclusions:**

The formulation of ME combined with the penetration enhancer can effectively improve the solubility and percutaneous absorption efficiency of acacetin, providing a new option for the non-invasive delivery of acacetin.

## Introduction

Acacetin, a natural flavone first found in *Saussurea involucrata* (Kar. et Kir.) Sch.-Bip (Asteraceae), was reported to possess the beneficial effects of anticancer (Jung et al. 2014; Kim et al. 2014) and anti-inflammation (Huang and Liou 2012; Kim et al. 2012; Carballo-Villalobos et al. 2014). In the previous study, we demonstrated that acacetin could act as a potential drug candidate for the treatment of atrial fibrillation (AF) by multiple atrial-specific ion channel blocking mechanisms (Li et al. 2008; Wu et al. 2011, 2013) and prevent the induction of experimental AF in anesthetized canines after duodenal administration (Li et al. 2008). The median lethal dose (or 50% lethal dose, LD_50_) calculated by the Bliss method (Rosiello et al. 1977) was determined as 721.7 mg/kg in mice after administrating a single dose of acacetin prodrug through the tail vein. Compared with currently market-available drugs for the treatment of AF, such as antiarrhythmic drugs (Ravens and Christ 2010), acacetin has evident advantages such as reduced proarrhythmic effects and negligible toxicity resulting from high atrial selectivity. However, acacetin has the common disadvantages of flavone compounds, such as very poor water solubility (64.4 ± 10.9 ng/mL), limited ethanol solubility (0.712 ± 0.002 mg/mL), and extremely low and unregular oral bioavailability in beagle dogs (6.37 ± 8.35%, *n* = 3). In recent studies, we have prepared a highly water-soluble phosphate prodrug of acacetin for first aid, which exert an effective termination of experimental persistent AF induced by atrial triggering with vagal nerve stimulation in beagle dogs by infusion. The therapeutic window was determined as 61.1 ∼ 650 ng/mL by the correlation analysis of pharmacodynamics and pharmacokinetics. However, in general terms, drug delivery aims to achieve efficient pharmacokinetics together with no or minimal invasiveness. Thus, it’s still necessary to develop a novel formulation with good clinical compliance and longer therapeutic plasma concentration for daily use.

Transdermal drug delivery system (TDDS) is preferred for drugs with significant first-pass effect from the standpoint of translation medicine research and clinical application. TDDS could provide a prolonged period of administration, during which plasma concentrations could be maintained within the therapeutic window, and avoid the first-pass effect for compounds with short biological half-life. However, skin is only permeable to a small number of molecules of a few hundred Daltons and an appropriate octanol-water partition coefficient (Wiedersberg 2014; Marwah et al. 2016). To achieve efficient transdermal drug delivery, it is presumed that a drug delivery system with a higher concentration (Lopes 2014; Sintov 2015) and smaller size would help (Liuzzi et al. 2016). Microemulsions (ME) have been proven as a highly promising alternative system for dermal and transdermal delivery by virtue of their nano-size, higher solubilization tendency for hydrophobic drugs, robust formulation advantages, and easier scalability in the industrial milieu (Santos et al. 2008; Singh and Pai 2015).

In this study, we investigated the feasibility of developing a novel microemulsion (ME) preparation for the transdermal delivery of acacetin. ME formulations were designed, optimized and evaluated comprehensively. Pseudo-ternary phase diagrams and the simplex lattice experiment design were employed in the process of formulation design and optimization. The effects of enhancers on the permeability of ME were evaluated. The *ex vivo* permeation behavior and *in vivo* pharmacokinetics in rats were conducted to estimate the feasibility of acacetin-ME for transdermal delivery. By the application of ME and appropriate enhancers, we have demonstrated an increased solubilization capability as well as enhanced permeability for acacetin.

## Materials and methods

### Materials

Acacetin was synthesized by Yaomingkangde Pharmaceutical Company Limited. (2-Hydroxypropyl)-®-cyclodextrin, PEG300, PEG400, RH40 Cremophor® (RH40), Cremophor®EL (CrEL), oleic acid, isopropyl myristate (IMP) and Tween 80 were purchased from Sigma; Labrafac Lipophile WL 1349(1349), Labrasol® (Labra), Transcutol® (Trans), lipoid MCT were generous gifts from Gattefosse (Saint-priest, France); mineral oil, ethyloleate were purchased from Advance Company (Hong Kong, China). Methanol and acetonitrile were of chromatographic grade. All of the other chemicals and reagents used were of analytic grade.

### Solubility study of acacetin in oils, surfactants, cosurfactants and other solubilizer

Solubility studies were conducted to select the appropriate components of ME formulation with high drug loading capacity. In the process, an excess amount of acacetin was separately added into 0.1 g of solvents such as ethanol, PEG300, PEG400; oils such as Lipoid MCT, mineral oil, IMP; surfactants like CrEL, Tween 80, Labra; co-surfactants like Trans. Then samples were vortexed for 5 min thoroughly mixed and followed by constantly shaken at 37 °C for 24 h to achieve the dissolution equilibrium. After that, the samples were centrifuged at 14 000 rpm for 30 min to precipitate the un-dissolved particles. The concentration of acacetin in the supernatants was qualified by HPLC analysis. All experiments were performed in triplicate.

The HPLC system was equipped with a C-18 column (Grace, 4.6 × 150 mm, 5 μm) and mobile phase was composed of a mixture of water and methanol (v/v, 3:7, with 0.2% phosphate acid) using an isocratic elution (Hitachi L2130 pump, L2200 Autosampler and L2400 Detector, Hitachi, Japan). The flow rate was 1.0 mL/min. The UV absorbance detector was set at 330 nm.

### Construction of pseudo-ternary phase diagrams

On the basis of solubility study and HLB value, RH40 was selected as surfactant, 1349 and MCF-NF were selected as oil phase, PEG300 and Trans as co-surfactants. Pseudo-ternary phase diagrams were constructed by water titration method (Sintov and Botner 2006). The weight ratio of surfactant/co-surfactant (km) was first set as 2:1. Then a series of self-emulsifying systems composed of oil and Smix (surfactant/co-surfactant) were prepared with the weight ratio of oil to Smix ranging in 9:1, 8:2, 7:3, 6:4, 5:5, 4:6 3:7, 2:8, and 1:9. The freshly prepared self-emulsifying systems were titrated with distilled water under magnetic stirring at room temperature (R.T.), respectively. These systems were examined visually and carefully after each addition of distilled water. The ending point was recorded as the solution was changed to cloudy or opaque from transparent. The weight percentage of each component was calculated to construct pseudo-ternary phase diagrams by Origin8.0 software. The ME region (A_T_) was marked and calculated by Vimage 2014 (Vezu Tech CO., LTD.). After the formulation of ME was determined, the weight ratio of km was varied from 1:3 to 3:1. At each specific km, the pseudo-ternary phase diagrams were constructed by water titration method as mentioned above, and also the areas of A_T_ were compared.

### Simplex lattice experiment design

In this study, a simplex lattice experiment design was introduced to screen and optimize the composition of ME formulation (Zhu et al. 2008). The concentrations of oil (X1), Smix (X2), and water (X3) were opted as independent variables while particle size (Y1), polydispersity index (PI, Y2) and equilibrium solubility of acacetin (Y3) as dependent variables. In the design, the total concentration of oil, Smix and water was kept constantly 100% while the ratio of the three mixture factors was altered within the specific realm. The relationship between independent variables and dependent variable was analyzed with Design-Expert 8.0 as well as the experiment design displayed in Table 1. In consideration of smaller particle size and PI, higher solubilizing capacity, optimal formula would be recommended.

**Table 1.** The formulation designed based on simplex lattice model and the response results.

	Oil	Smix	Water	Size (nm)	PI	Solubility (mg/g)
1	0.048	0.357	0.595	34.2	0.126	741.6
2	0.280	0.420	0.300	162.4	0.907	657.4
3	0.167	0.250	0.583	181	0.259	237.3
4	0.020	0.280	0.700	33.9	0.383	263.5
5	0.034	0.637	0.330	0	2	1658
6	0.120	0.180	0.700	305.6	0.287	155.9
7	0.138	0.364	0.498	119.5	0.538	602.4
8	0.280	0.420	0.300	125	0.42	657
9	0.034	0.637	0.330	0	2	1658
10	0.020	0.280	0.700	21.8	0.525	265
11	0.165	0.453	0.382	0	2	573.8
12	0.131	0.569	0.300	0	2	1216
13	0.131	0.569	0.300	0	2	1218
14	0.026	0.490	0.484	189.4	0.507	669.5
15	0.120	0.180	0.700	311.1	0.308	146.6
16	0.224	0.336	0.440	133.1	0.229	290.4

### Preparation of acacetin-loaded ME

According to the established optimal formula (12.2% MCF-NF, 39.8% Smix and 48% water), the corresponding amount of each component, e.g., oil, surfactant and co-surfactant were weighed out exactly and blended together adequately. Then measured amount of acacetin was dissolved into very small amount of dimethyl sulfoxide (DMSO) and added into the oily mixtures. Ultimately, a weighed quantity of distilled water was added into the system drop by drop under mild magnetic stirring for 5 min. The whole process was performed at R.T. The particle size of acacetin loaded ME formulation were measured by Dynamic Light Scattering at 25 °C using Malvern Zetasizer Nano-ZS (Malvern Instruments Ltd., Worcestershire, England). All experiments were performed in triplicate.

### Ex vivo permeation study

Preparation of skin: Skin permeation study was carried out according to the National Institute of Health Guide for Care and Use of Laboratory Animals (1996) and was approved by the Institutional Animal Care and Use Committee of Changzhou University (NO. Y20210019). Male Sprague-Dawley (SD) rats weighing about 250 g were obtained from Laboratory Animal Unit of University of Hong Kong. The rats were housed in cages and supported with standard laboratory diet and water. Before the experiment, the rats were fasted overnight. The abdominal skin was excised surgically after rats were anesthetized by pentobarbital and the hair on the skin was shaved with electrical shaver. Then the subcutaneous tissues of skin were removed. The obtained skin was washed with normal saline and stored at −80 °C. The skins were inspected for integrity before further use to ensure that the samples were free from surface defects (Ustundag Okur et al. 2011; Zhao et al. 2014).

Permeation experiment: In order to evaluate the permeability of the prepared formulation or the drug itself, the *ex vivo* permeation study was conducted with Franz diffusion cell and excised mouse skin as a permeable model (Yuan et al. 2006). All skin samples were defrosted immediately prior to use. The effective penetration area and receptor cell volume were 1.71 cm^2^ and 15 mL, respectively. The receptor compartment was filled with 15 mL of phosphate buffer solutions (0.1 M, pH 7.4) containing 1% sodium dodecyl sulfate (SDS) to maintain sink condition. The whole system was kept at 37 °C and under magnetic stirring at 600 rpm throughout the entire process. Caution was taken to remove all air bubbles between the underside of the skin and the receptor solution. Then, 0.5 mL of acacetin-loaded Mes to be tested or other samples, all containing an equal amount of drug, e.g., 5 mg, was given to the stratum corneum (SC) side of the skin, respectively. Afterwards, the donor compartment was sealed with paraffin film to prevent water evaporation from the system. For each experiment, 0.3 mL of the sample was taken out from the receptor compartment at pre-determined time point and replenished instantly with an equal volume of fresh receiver medium. All samples were treated with 10 times volume of methanol, vortexed for 2 min and then filtered through 0.22 μm membrane filters before HPLC analysis. The cumulative amount of acacetin that permeated through the excised skin (Qt, μg/cm^2^) was calculated based on the reported methods (Zhu et al. 2009). Maximum flux across the skin (Jss, ng/cm^2^/h) was calculated from the slope of the Qt-time graphs. Apparent permeability coefficient (*P*app, cm/h) was calculated by the following equation:

Papp = Jss/Cd

Where Cd is acacetin concentration in the donor compartment (1.0%, wt%).

The maximum flux of acacetin and acacetin-prodrug were also evaluated by dissolved in 50% Tween 80.

### Pharmacokinetic study

Pharmacokinetic: Rats were divided into 3 groups (*n* = 3) for transdermal administration. The rats were anesthetized by ip 50 mg/mL pentobarbital and the upper back region was very carefully shaved with an electric hair clipper to keep the integrity of the shaved skin. 200 μL of the transdermal formulations (containing an equal amount of drug 2 mg) was applied every 1 h within 6 h, and the reservoir was covered with Tegaderm film (3 M Co., St. Paul, MN, USA), in order to prevent the possible solvent evaporation. Blood samples 0.5 mL were collected at 0, 60, 120, 180, 240, 360, 480 and 1440 min from the orbital venous plexus at predetermined-time point over 24 h after formulation application. Plasma was separated by centrifuged (5000 rpm, 10 min) and frozen at −80 °C until analysis.

HPLC-MS analysis: Firstly, a set of plasma standards of acacetin were prepared by mixing of different amount of acacetin with 100 μL of blank rat plasma to give final acacetin concentrations of 0.5 ∼ 1000 ng/mL in plasma, respectively. Then an aliquot (1 mL) of the internal standard (IS) solution (30 ng/mL pentamethylquercetin in methanol) was added to each plasma standards. After vortexed mixing for 5 min to precipitate plasma protein, followed by centrifugation at 12,000 *g* for 30 min. The final supernatant was injected into UPLC/MS/MS system for analysis. In addition, three quality control samples (QCs) at 1, 25, and 400 ng/mL were prepared in blank plasma. Secondly, frozen serum samples were thawed at room temperature (R.T.). The post-treatment of plasma samples was the same as plasma standards as described above.

An UFLC system (Shimadzu, Tokyo, Japan) coupled with a tandem mass spectrophotometer system (AB Q-Trap 4500, AB Sciex, USA) was used for the quantitative analysis of acacetin. Chromatographic separations were achieved on an RP C18 column (Kinetex, 2.1 × 150 mm, 2.6 μm) at 40 °C. The gradient mobile phase consisted of 5 mM ammonium formate containing 0.1% formic acid (A) and acetonitrile (B) was pumped at a flow rate of 0.4 mL/min. The initial mixture (90 A:10B) was maintained for 0.4 min; mobile phase B was increased to 90% at 1.9 min and held at 2.5 min. The mixture returned to initial conditions at 2.6 min, followed by 1.9 min of equilibration. The total run time was 4.5 min. The sample injection volume was 1 μL. The analytical method was validated according to the FDA guidelines for bioanalytical method validation (Tiwari and Tiwari 2010). Mass spectrometric data were acquired in positive electrospray ionization mode with the following source parameters: ion Spray voltage, 5500 V; temperature, 550 °C; curtain gas, 25; ion source gas 1, 45; and ion source gas 2, 45. Data were recorded in multiple reaction monitoring modes (MRM). The mass transitions chosen for the quantitation of the compounds were *m/z* 285.0→*m/z* 241.8 for acacetin and *m/z* 373.0→*m/z* 312.0 for the IS. The retention time of IS and acacetin was 1.97 and 2.02 min, respectively.

Linearity range with 1/x/x weighting was from 1 ∼ 500 ng/mL. The lower limit of quantification was 0.5 ng/mL, and the limit of detection was 0.1 ng/mL for acacetin. Assay accuracy at low, medium, and high QCs was 100 ± 3.18%, 104 ± 2.89% and 97.5 ± 5.12%, respectively (*n* = 5). Samples were stable after 4 h on the autosampler.

Statistical data analysis: Data reported were expressed as the mean ± SEM (*n* = 3). The statistical analysis of the data was carried out using a one-way analysis of variance (ANOVA) with *p <* 0.05 as the minimal level of significance unless otherwise indicated.

## Results

### Solubility study

**Figure 1.**
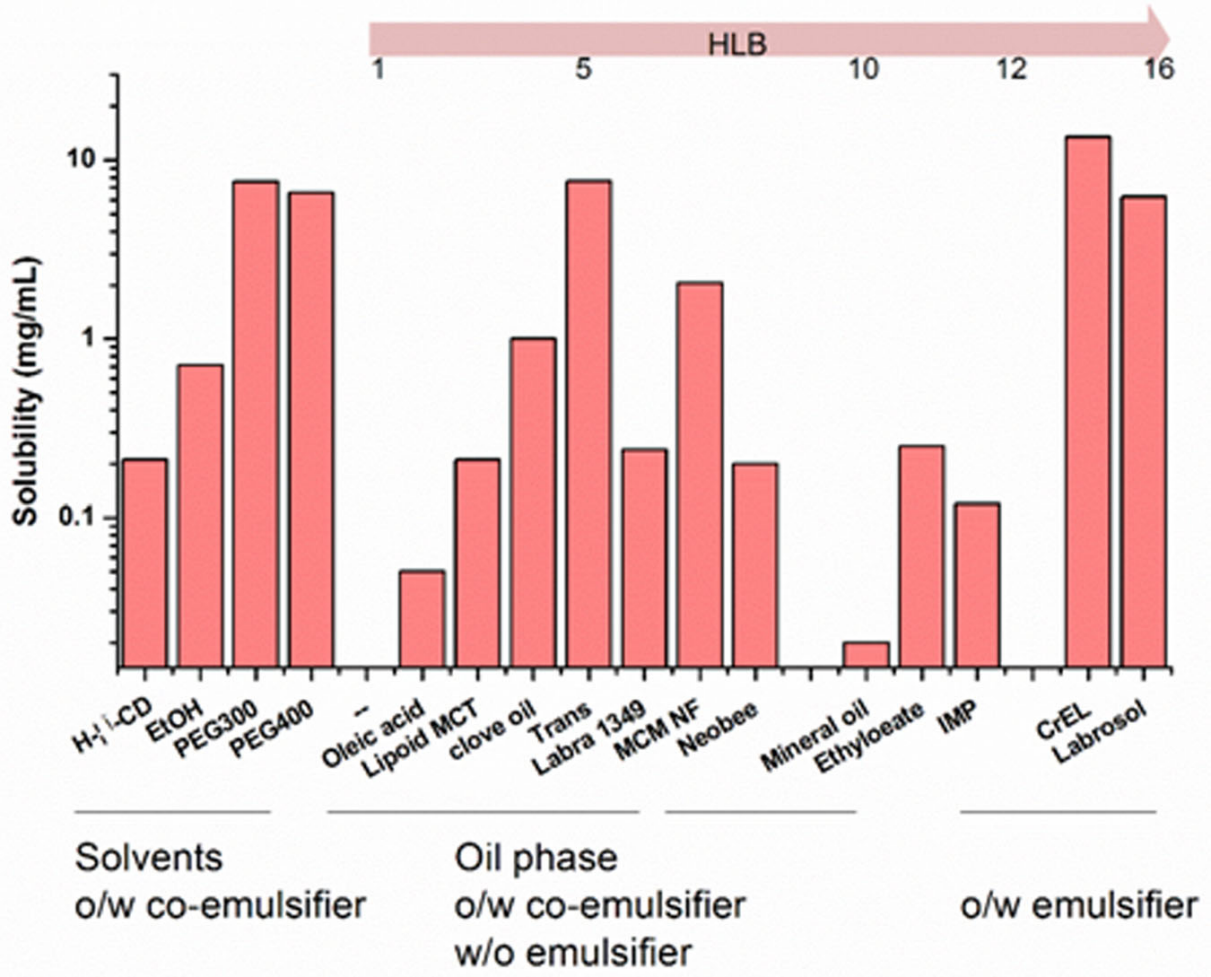
Acacetin solubility in selected oils, surfactants and co-surfactants.

The equilibrium solubility of acacetin in various vehicles is shown in Figure 1. RH40 presented the highest solubility of acacetin (13.5 mg/mL) in the screening realm and was opted as the surfactant of ME formulation. PEG300 and Trans were selected as co-surfactants for further investigations due to the similar higher solubilizing capacity and different potential permeation-enhancing effects. 1349 and MCF-NF were selected as the oil phase.

### Construction of pseudo-ternary phase diagrams

**Figure 2.**
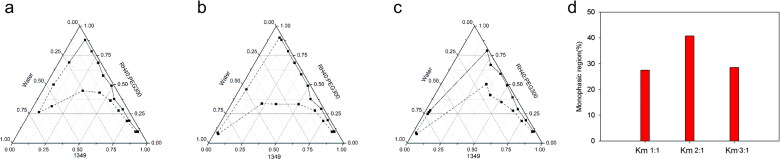
Pseudo-ternary phase diagrams consisted of 1349 as oil, RH40/PEG300 as Smix (a 1:1, b 2:1, c 3:1, the solid and dashed line represent the starting and ending point of the titration respectively); d comparisons of monophasic region.

**Figure 3.**
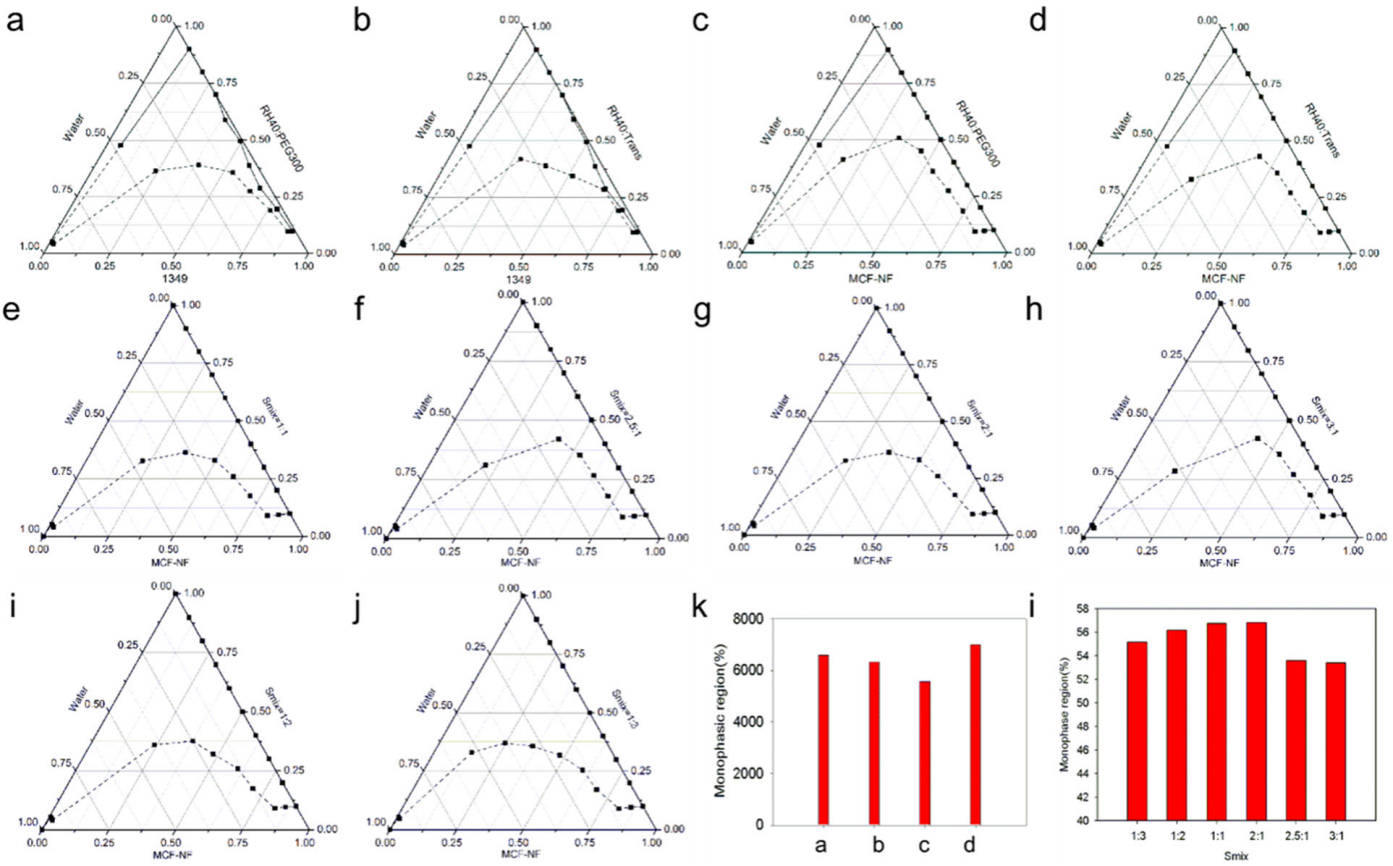
Pseudo-ternary phase diagrams of ME consist of 1349 (a, b) and MCF-NF (c, d, e - j) as oil, RH40/PEG300 as Smix (a, c) and RH40/Trans as Smix (b, d, e - j); (k, l) variation of the total monophasic region as a function of the surfactant to cosurfactants ratio in the system designed ME system.

On the basis of preliminary screening results of the solubility study, 1349, PEG300 and RH40 were firstly employed as oil phase, co-surfactant and surfactant. The Km was set as 1:1, 2:1 and 3:1. The pseudo-ternary phase diagrams were drawn and A_T_ were compared (the solid and dashed line represent the starting and ending points of the titration respectively). As seen in Figure 2, the Km 2:1 could yield a bigger A_T_. Thus, we fixed Km as 2:1, 1349 and MCF-NF as oil phase, PEG300 and Trans as co-surfactant, respectively. As compared in Figure 3(a–d) and k, MCF-NF and Trans were finally determined as oil phase and co-surfactant. After that, the range of Km was varied from 1:3 to 3:1. The A_T_ were compared and listed in Figure 3(e–g) and I. Thus, the system consisting of MCF-NF and RH40:Trans = 2:1 was supposed to have better miscible ability with water for further optimization.

### Optimization of ME formulation

A simplex lattice experiment design was utilized to screen and optimize the ME formulation subsequently. As a higher solubility warrants the drug loading capacity in the optimized formulation and a smaller particle size is supposed to promote drug release and transport through membranes. Based on the results from pseudo phase graphs, the range of independent variables was selected as follows: for Oil (X1, MCF-NF, 1.5 ∼ 28%), Smix (X2, RH40:Trans = 2:1, 11.8 ∼ 66.7%) and water (X3, 30 ∼ 70%). A simplex lattice experiment design containing 16 runs generated by Design-Expert 8.0 software and corresponding response were presented in Table 1.

**Figure 4.**
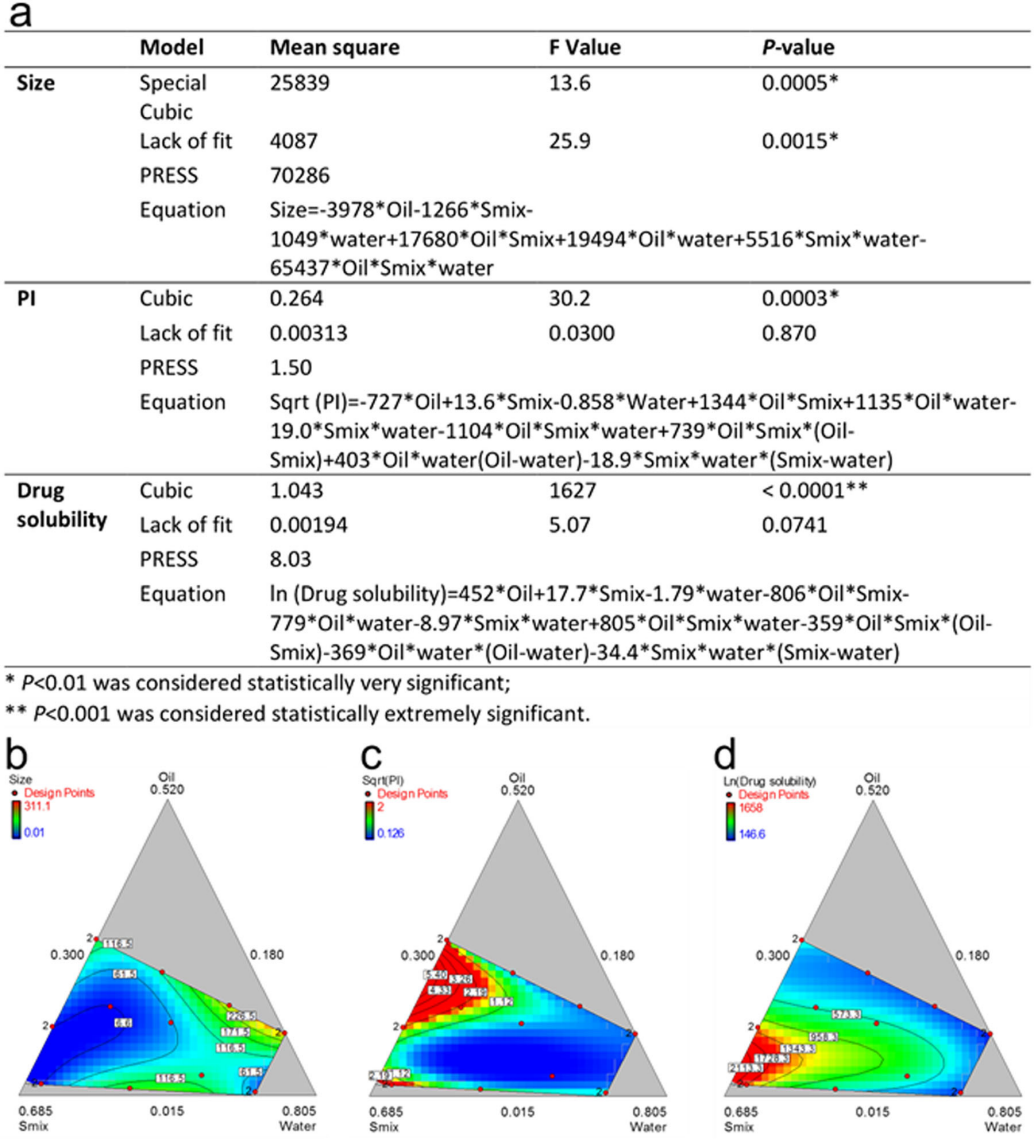
Reduced regression results of the observed response (a); contour plot of the effect of independent variables on the size (b), PI (c) and drug solubility (d).

Hereafter, analysis and optimization of the obtained data were conducted. In the process of fit summary, the regression calculations were carried out to fit all of the polynomial models such as linear, special cubic and full cubic polynomials to the selected response. It produced statistics like *P*-values, lack of fit and PRESS values for comparing the models so as to select statistically significant models for the desired response, such as the smaller size and PI, higher solubility. After ANOVA analysis of the suggested models obtained from the aforesaid process, the models that possessed the smallest *P*-values and PRESS values were built for PI and solubility, as shown in Fig. 4a. The contour diagrams were drawn and list in Fig. 4b–d, to visually depict the correlation between responses and different ratios of X1, X2 and X3.

The optimal ME formulation comprised of 12.2% MCF-NF, 39.8% Smix and 48% water was recommended and selected. Acacetin loaded ME with particle size 36.0 ± 3.6 nm, PI 0.337 ± 0.023 and drug solubility 803.7 ± 32.1 mg/g was prepared thereafter.

### Ex vivo permeation study

**Figure 5.**
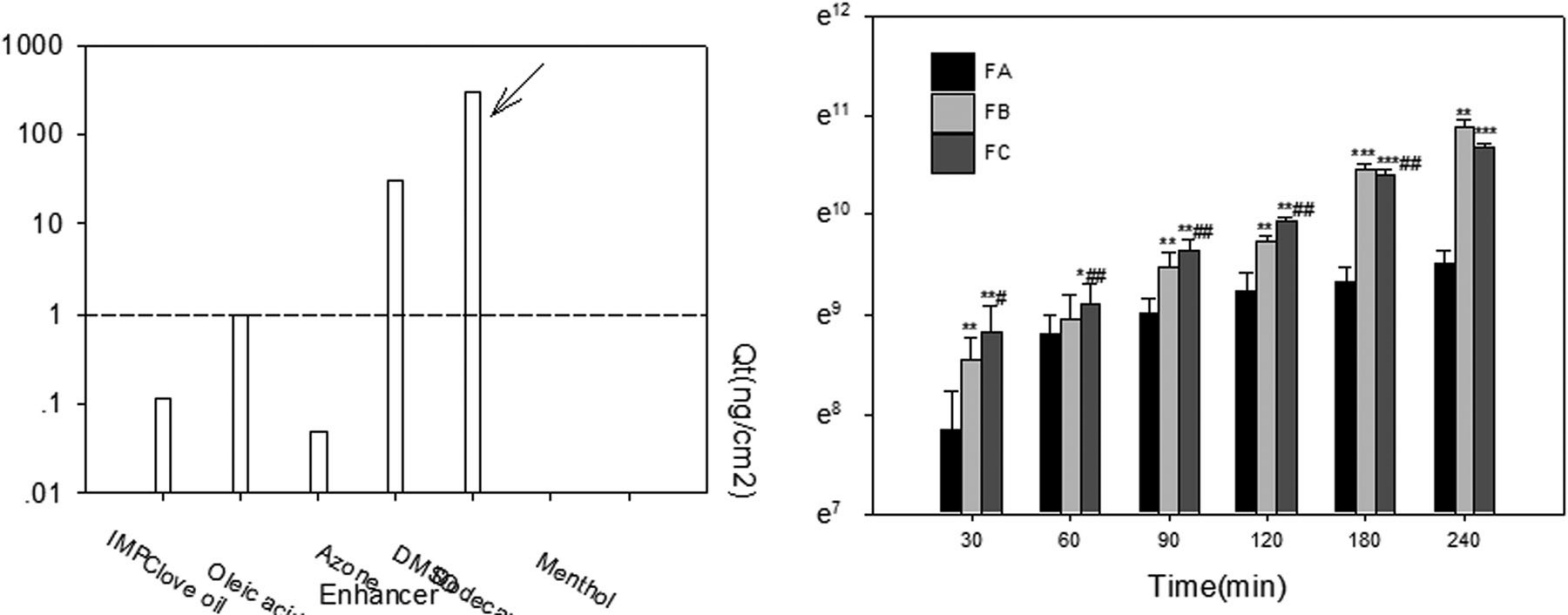
(a) The solubility of acacetin in different CPEs; (b) *ex vivo* permeation profiles of acacetin from three designed formulations. *p < 0.05, **p < 0.01, ***p < 0.001 statistically significant compared with FA; ^#^*p* < 0.05, ^##^*p* < 0.01 statistically significant compared with FB.

The skin is impermeable to most molecules and the SC is generally considered to be the main barrier, due to its unique composition of laterally overlapping, quasi-columnar stacks of corneocytes embedded in intercellular lipid multi-layers. Chemical penetration enhancer (CPE) in the formulation is generally employed to overcome SC barrier and achieve therapeutically plasma concentration by transdermal delivery (Diblíková et al. 2014). The drug solubility in CPE could act as a useful starting point to guide the selection of appropriate CPE. As shown in Fig. 5a, azone and DMSO presented higher solubility. However, the azone was excluded for the following reasons: (1) azone is preferred for water soluble drugs as CPE (Jampilek and Brychtova 2012); (2) limited enhancing permeability for acacetin in preliminary studies. Hence, DMSO was studied since it had been approved for enhancing the permeability in topical formulations from 2009 (Marren 2011).

In addition, the compatibility between CPE and drug should be considered, such as if the skin residence time of CPE is very different from the drug (Alexander et al. 2012). In the light of preliminary optimization by the simplex lattice experiment, to achieve a balance between enhanced permeation and less skin irritation, the *ex vivo* permeability and *in vivo* pharmacokinetic studies were conducted and compared in recommended ME formulation (FA), FA with 10% DMSO (FB) and solubilizing formulation with 50% DMSO (FC). The formulation FC was composed of 50% DMSO, 30% CrEL, 10% ethanol and 10% PEG300. And the drug concentration was determined as 10 mg/mL in all formulations to be tested.

*Ex vivo* permeation studies were conducted to compare the skin permeation ability among formulations of FA, FB and FC. Cumulative amount of acacetin permeated (Qt) percutaneously were depicted in Figure 5(b). Compared with FA, FB (FA with 10% DMSO) exhibited very significant differences (*p <* 0.01) at *T* = 30, 90, 120, 240 min, and extremely significant differences (*p <* 0.001) at *T* = 180 min. These results demonstrated that enhanced skin permeability of ME could be achieved by 10% DMSO significantly. High percent of DMSO (50%) acting as CPE in FC was verified at every time point, especially before 120 min, compared with FA and FB.

### Pharmacokinetic study

**Figure 6.**
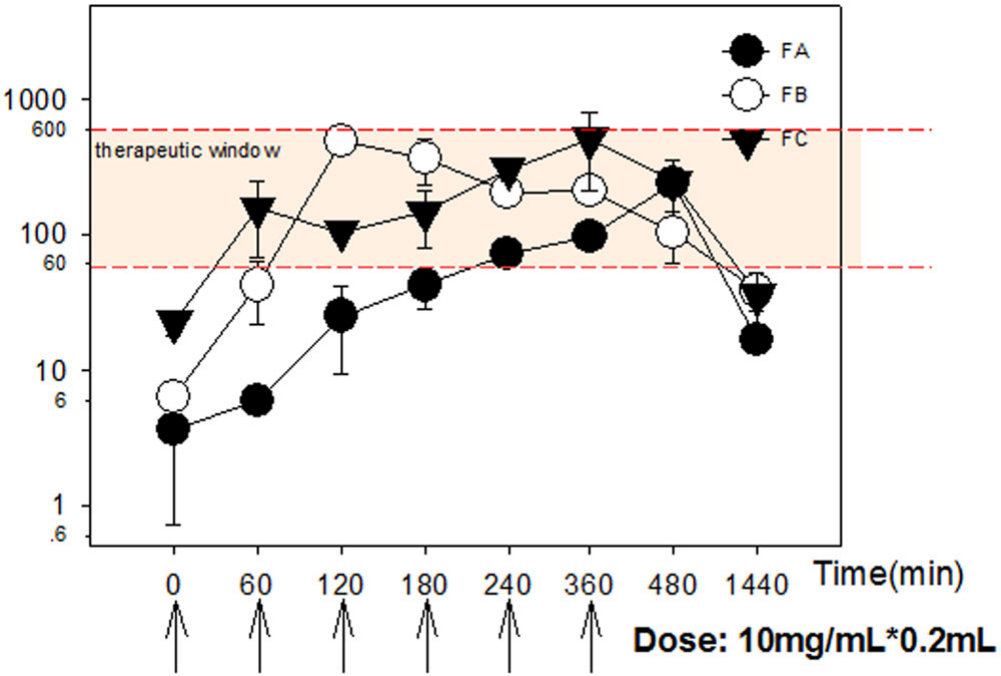
Mean plasma concentration profiles of acacetin after transdermal administration of various formulations. Data were expressed with Mean ± SEM (*n* = 3).

The mean acacetin plasma concentration-time profiles after transdermal administration are shown in Figure 6. Pharmacokinetic parameters such as maximal plasma drug concentration (Cmax) and time to maximal plasma drug concentration (Tmax) were read directly from individual plasma concentration-time profiles. The duration time of action was analyzed and obtained by correlation with the therapeutic window (61.1 ∼ 650 ng/mL obtained in experimental persistent AF). The area under the concentration-time curve from time 0 to t (AUC_0-t_) was calculated by the linear up-log-down rule. As shown in Table 2, FB exhibited considerably higher Cmax and improved bioavailability in comparison with FA. The AUC_0-t_ of FC is slightly higher than FB, but it’s not significant due to big variation. FC presented a faster permeation rate than FA and FB especially at the early post-administration time points. These results are in good agreement with the *ex vivo* permeability data.

**Table 2.** The permeation and pharmacokinetic parameters of Acacetin after transdermal administration of different formulations at a dose of 8 mg/kg every 60 min within 360 min (*n* = 3).

Formulation	Percentage of DMSO	Jss (ng/cm2/h)a	Permeability coefficient (Papp, ×106, cm/h)b	Tmax (h)c	Cmax (ng/ml)d	Duration of action(min)e	AUC0-t (min*μg/ml)f	AUC0-∞ (min*μg/ml)g
FA	No	47.0 ± 1.61	4.7 ± 0.16	8	241.5 ± 114.5	240 ∼ 1000	112.8 ± 34.2	121.4 ± 33.1
FB	10%	233.4 ± 16.0	23.3 ± 1.6	2	468.3 ± 51.2	60 ∼ 1000	174.9 ± 7.8*	196.6 ± 11.0*
FC	50%	181.3 ± 0.729^#^	18.1 ± 0.0729^#^	6	563.2 ± 230.7	26 ∼ 1200	217.0 ± 71.6*	234.1 ± 77.3*

*p < 0.05 statistically significant compared with FA; ^#^*p* < 0.05 statistically significant compared with FB.

^a^Maximum flux across the skin calculated from the slope of the Qt-time graphs

^b^Apparent permeability coefficient calculated by **Jss**/Cd, in which Cd is Acacetin concentration in the donor compartment (1.0%, wt %).

^c^Time of peak concentration.

^d^Peak of maximum plasma concentration.

^e^Time maintained above the therapeutic plasma concentration.

^f^Area under the concentration time profiles curve until last observation.

^g^Area under the concentration time profiles from time 0 to infinity.

In addition, therapeutic plasma concentration could be maintained within 60 ∼ 1200 min and 26 ∼ 1000 min in FB and FC group, compared with FA within 240 ∼ 1000 min. The earlier onset of effective time in the FC group certified enhanced permeation effects of high percent DMSO into the systemic circulation. The comparable duration time of action, AUC_0-t_ and AUC_0-∞_ between FB and FC groups certified the promising feasibility of a combination of ME and DMSO (FB) to be employed for the transdermal delivery of lipophilic drugs (Vitorino et al. 2013).

## Discussion

Owing to the extremely low oral bioavailability of acacetin and short biological half-life, the development of TDDS is considered. Given that the skin’s principal function is to act as a protective barrier, and that the rate of molecular transport through (in particular) the outermost layer, the SC, is highly constrained and very slow. Therefore, the evaluation of the molecule’s skin penetrability by maximum flux across the skin (Jss, ng/cm^2^/h) is the default starting position. The permeability of acacetin prodrug was compared with the parent drug. As listed in Table 3, both the physical-chemical parameters and the results from *ex vivo* permeability studies verified that parent water-insoluble acacetin is a good candidate compared with water soluble acacetin prodrug. Thus, the parent acacetin was selected and studied thereafter.

**Table 3.** Physic-chemical parameters of Acacetin and phosphate prodrug of Acacetin.

	Parent Acacetin	Phosphate prodrug of acacetin
Chemical structure	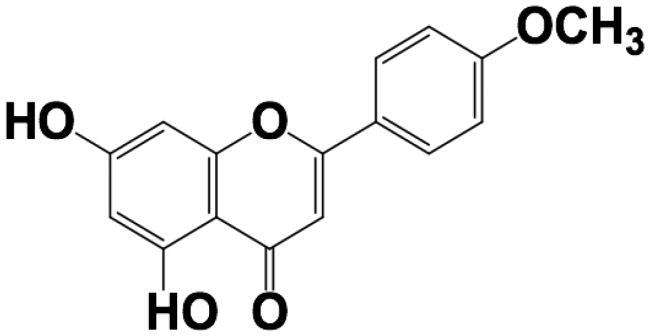	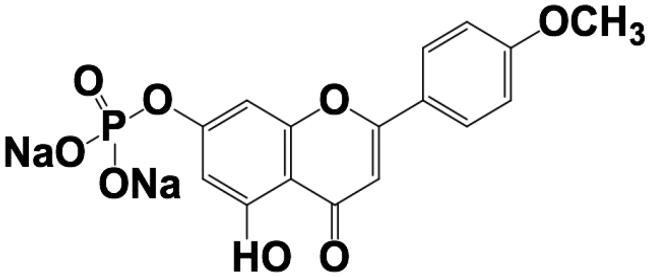
Molecular weight	284.26	408.21
Water solubility at R.T.	64.4 ± 10.9 ng/mL	126.4 ± 2.47 mg/mL
logP	3.4	−0.7
*Ex vivo* skin permeability(Jss, ng/cm^2^/h)	2224.3 ± 27.8 ng/cm^2^/h	Lower than detection limit
Permeability coefficient(*P*app, ×10^6^, cm/h)	22.2 ± 0.378	–

It is believed that the higher drug solubility in ME could enhance the skin permeation potential (Zhao et al. 2006; Hathout et al. 2010), driven by higher concentration grades. In this study, the equilibrium solubility of acacetin in various vehicles was conducted to evaluate the compatibility between acacetin and different solvents. RH40 was opted as the surfactant; PEG300 and Trans were selected as co-surfactants. 1349 and MCF-NF were selected as the oil phase. In addition, to determine if acacetin could be effectively solubilized by β-CD inclusion complex, we also tried to solubilize acacetin by HP-β-CD. However, the preliminary solubility data was not as satisfactory as expected (0.21 mg/mL).

The pseudo-ternary phase diagrams were then drawn by the selected surfactant, co-surfactants and oils by water titration method. The system consisted of MCF-NF and RH40:Trans = 2:1 was supposed to have better miscible ability with water for further optimization by a simplex lattice experiment design with the goal of higher solubility and smaller particle size to achieve quick drug release and transportation through membranes. From the perspective of PI, the smaller PI could reveal (1) the homogeneous state of ME size, (2) the certainty of the bicontinuous phase and discontinuous phase.

However, we didn’t obtain a significant model for size. It is presumed that with the water adding during the titration the type of emulsion will gradually change from *w/o* to *o/w*. The effect of formulation on the size is not a linear process or any process could be described by some known mathematical models. As shown in Figure 4(b), the dynamic changes of the designed response are irregular. To the best of our knowledge, it is still challenging to characterize the tiny structure of ME.

The skin has evolved to prevent excessive water loss from the internal organs and to limit the ability of xenobiotics and hazardous substances to enter the body. Various strategies have been developed for the enhancement of skin permeability by such as (1) altering physicochemical properties of SC; (2) by changing a hydrating property of SC; (3) by altering structure of lipids and protein in intercellular channel *via* carrier mechanism (Csizmazia et al. 2012). Due to the high percentage components of surfactant and co-surfactant, ME could decrease the skin barrier function and consequently its electrical resistance and result in the high permeation fluxes (Kogan and Garti 2006; Hathout and Elshafeey 2012). Thus, ME was widely designed for topical or transdermal delivery. Besides, the application of chemical penetration enhancer (CPE) in the formulation might help. The overall effects of CPE on the skin barrier may best be explained by a Diffusion-Partition-Solubility theory. Within passive methods, the combined application of ME and chemical enhancer might show advantages.

*Ex vivo* permeation studies were conducted to compare the skin permeation ability among formulations of FA, FB and FC. Enhanced skin permeability of ME could be achieved by 10% DMSO (FB) and 50% DMSO(FC) at every time point. The enhanced permeability mechanisms of DMSO might due to (1) DMSO is a polar aprotic solvent able to dissolve polar and nonpolar small molecules, fluidize skin lipid molecules for vesicles permeation (Simoes et al. 2016), it also increases the entrapment of several drugs in lipid carriers by enhancing their solubility (Farooqui et al. 2022); (2) DMSO may also enhance transdermal penetration by improving partitioning of drug into skin layers (Omar et al. 2019). After 120 min, FB showed some advantages compared with FC, which probably attributing to improved skin hydration during transportation (Santos et al. 2008; Hathout and Elshafeey 2012). There should keep balance between enhancing the permeability and hydration of the skin stratum corneum (Fouad et al. 2013; Todosijević et al. 2015).

The results from *in vivo* pharmacokinetic analysis of the FA, FB and FC are in good agreement with the *ex vivo* permeability data. FB with DMSO added as CPE could improve skin permeability of ME to a small extent (10%) and reduce the occurrence of skin irritation and systemic adverse effects involved (Marren 2011). So, the combined application of ME and DMSO offers many advantages in transdermal drug delivery, such as a higher solubilization capacity, enhanced permeability and reduced irritation. Our results demonstrated that by mixture with ME, the amount of DMSO acting as CPE could reduce to 10% from a high percent (50%).

## Conclusion

In the present study, acacetin-loaded ME formulations were successfully developed and evaluated for transdermal delivery. Pseudo-ternary phase diagrams were employed to depicted the ME regions for the screening of the ME ingredients. The formulations were further employed with a simple lattice experiment design. The optimized formulation FA showed an average particle size of 36 nm. In *ex vivo* permeation study, FA with 10% DMSO showed significant higher cumulative amounts of acacetin permeated after 4 h application than FA itself. It can be concluded that ME is a potential carrier for the transdermal delivery of acacetin. Further work is required to determine whether ME may ultimately be incorporated into a transdermal patch and take advantage of the favorable delivery rate as well as the lower skin irritation potation.
